# Hospital Progress and Finance

**Published:** 1923-07

**Authors:** 


### HOSPITAL PROGRESS AND FINANCE.

### ROYAL INTEREST IN HOSPITALS.

THE Prince of Wales's recent tours have been remarkable for the number of hospitals that he has managed to visit, despite the fact that industrial centres and factories were the principal object in view. At Wolverhampton, where a special appeal coincided with his visit, at Leeds where he inspected the Ministry of Pensions Hospital, Beckett's Park, and immediately on his return to London where he presided at the annual meeting of King Edward's Hospital Fund, his interest in the voluntary system was conspicuous, and few, if any, public claims on his time exceed those of hospital work. It is only in the past fifty years that the Royal Family has so identified itself with hospital interests. King Edward when Prince of Wales may be said to have established the relation, and if we take it as a matter of course to-day, it is well to remember that the voluntary system only earned this recognition in the time of the Prince's grandfather.

### HOSPITAL SUBSCRIPTIONS AND INCOME TAX.

THE LORD MAYOR of Manchester, who is trying to raise a Two Million Shilling Fund for the Manchester Hospitals, inquired of Somerset House whether the subscriptions of firms in the city to his fund would be regarded as a trading expense in the assessment of Income Tax. He has received the following interesting reply: "The Board of Inland Revenue, for their part, would not normally be disposed to raise any objection to such allowances where the special contributions bear a reasonable relationship to past annual subscriptions and where they are necessary to meet a deficit incurred on current annual expenditure of the institutions in question. It is, of course, understood that the institutions are such as would be of direct benefit to the employés of the contributing firms. The General Commissioners for this district are, however, the competent authority in the first instance." This admission will not be lost on the Lord Mayor, and we await with interest the response of the General Commissioners of the district to the argument which he will doubtless press upon them. The Manchester fund needs every encouragement, for the city's contributions to hospitals compare very badly with those of other and smaller towns.

### PRIZES FOR HOSPITAL COLLECTORS.

A SOMEWHAT novel departure has been made by the Committee of the Carnarvonshire and Anglesey Infirmary, Bangor. To encourage holders of "extension fund" collecting boxes to make a special effort, the Committee offers four cash prizes, from £2 to 10s., for the best box collections of the year. We are familiar with the paid collector, who often develops into the salaried financial secretary. We are also acquainted with the collector who receives, or used to receive, a percentage on the total that he raised. But the cash prize for the four best box collections is more novel. It is an example of the commercialisation of every department of life which has often invaded, in different ways, the field of charity. It certainly deprives the collector of claiming personal service for his motive, though we believe that this, and not the prize, will in most cases determine the result. It remains to be seen if the new inducement is successful. If it is not, we at least shall not complain, since we are not much impressed with this half-way house between personal service voluntarily rendered and the frankly speculative sweepstake.

### A NEW ERA IN HOSPITAL SATURDAY?

THE vigour of the Hospital Saturday movement in the provinces could hardly be better shown than by the decision of the Coventry organisation to mark its jubilee. The Committee has determined if possible to raise £10,000 for the Coventry and Warwickshire Hospital over and above the sum which it presented last year. In other words, the movement proposes not only to continue its work of maintenance, but also to free the hospital from debt. This is both public-spirited and ambitious, and should startle the kindred organisations in such backward centres as Manchester into a sense of their shortcomings. The first meeting of the General Committee to receive the proceeds of the collection was held as long ago as October 27, 1874, so that Coventry was one of the first places to establish Hospital Saturday. Last year £14,500 was raised under the chairmanship of Mr. H. Warren, when Mr. T. H. S. Nichols, the present Secretary, completed twenty-six years' work. The result of this generous effort will be watched with interest, and if it succeeds, a new possibility of growth will be presented to all the Saturday Funds of the country.

### SALISBURY INFIRMARY LEAGUE.

THE Salisbury Infirmary League reports good progress in its work of multiplying the number of small weekly contributions. The membership is something like 10,000, and the educational Sunday meetings at which the organisation of the Infirmary is explained have been found particularly useful. Nevertheless, there is a large population which is not represented in the League, and there are certain villages and groups of villages perfectly well able to help which, for a variety of reasons, are unwilling to do so. In Salisbury itself many workers in small businesses and a number of domestic servants appear to be ignorant of the League and its objects, yet to the latter it should be particularly valuable, since, when ill, they have nothing but the Infirmary to fall back upon. For some obscure reason, it is easier to get hold of servants in the country. The League is clearly doing admirable work in the maintenance of an ancient institution which has suffered severely during the war, and needs the regular support of all who may themselves benefit by its help in time of need.

